# Effects of embryo energy, egg size, and larval food supply on the development of asteroid echinoderms

**DOI:** 10.1002/ece3.6511

**Published:** 2020-07-08

**Authors:** Stacy N. Trackenberg, Emily L. Richardson, Jonathan D. Allen

**Affiliations:** ^1^ Biology Department William & Mary Williamsburg Virginia USA; ^2^ Department of Biology East Carolina University Greenville North Carolina USA; ^3^ School of Biological Sciences Monash University Melbourne Vic. Australia

**Keywords:** asteroids, development, echinoderms, larvae, life history evolution, maternal investment

## Abstract

Organisms have limited resources available to invest in reproduction, causing a trade‐off between the number and size of offspring. One consequence of this trade‐off is the evolution of disparate egg sizes and, by extension, developmental modes. In particular, echinoid echinoderms (sea urchins and sand dollars) have been widely used to experimentally manipulate how changes in egg size affect development. Here, we test the generality of the echinoid results by (a) using laser ablations of blastomeres to experimentally reduce embryo energy in the asteroid echinoderms (sea stars), *Pisaster ochraceus* and *Asterias forbesi* and (b) comparing naturally produced, variably sized eggs (1.7‐fold volume difference between large and small eggs) in *A. forbesi*. In *P. ochraceus* and *A. forbesi*, there were no significant differences between juveniles from both experimentally reduced embryos and naturally produced eggs of variable size. However, in both embryo reduction and egg size variation experiments, simultaneous reductions in larval food had a significant and large effect on larval and juvenile development. These results indicate that (a) food levels are more important than embryo energy or egg size in determining larval and juvenile quality in sea stars and (b) the relative importance of embryo energy or egg size to fundamental life history parameters (time to and size at metamorphosis) does not appear to be consistent within echinoderms.

## Graphical abstract

In two species of sea stars, exogenous food levels are more important than maternal investment in determining larval and juvenile quality. The relative importance of maternal investment to fundamental life history parameters (time to and size at metamorphosis) does not appear to be consistent within echinoderms.

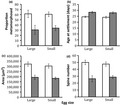

## 1 INTRODUCTION

Parents have limited resources to allocate to offspring, leading to trade‐offs between traits that may constrain the evolution of reproductive strategies (Van Noordwijk & de Jong, 1986; Williams, 1966). One such trade‐off occurs between the number and size of offspring that can be produced by a female in a single clutch (Roff, 1992; Sinervo, 1990; Stearns, 1992). Females that produce large clutches of small eggs have a higher fecundity compared to those that produce small clutches of large eggs; however, small eggs typically give rise to smaller offspring, which can have negative impacts on offspring fitness (Godfray, Partridge, & Harvey, 1991). For example, among fence lizards (*Sceloporus occidentalis*), small eggs hatch into smaller, slower juveniles while clutches of large eggs are smaller in number (Sinervo, 1990; Sinervo et al., 1992). Therefore, the fecundity advantage of large clutches can trade off against the fitness costs of smaller eggs, a well‐documented phenomenon that has been demonstrated in both laboratory manipulations and in wild populations (e.g., Sinervo et al., 1992).

Empirical examples of the trade‐off between the number and size of offspring can be found across a wide variety of taxa including plants, amphibians, birds, cladocerans, echinoderms, fish and reptiles (Arnold, 1992; Bernardo, 1996a; Emlet, McEdward, & Strathmann, 1987; Guisande, Sanchez, Maneiro, & Miranda, 1996; Landberg, 2014; Nager, Monaghan, & Houston, 2000; Sinervo, 1990; Venable, 1992). While vertebrates have formed the basis for many excellent studies of the egg size/number trade‐off, the widespread occurrence of parental care both before and after hatching (Bernardo, 1991; Monaghan & Nager, 1997; Mousseau & Fox, 1998) creates additional maternal effects that are difficult to quantify and can therefore confound experiments on offspring size effects (Bernardo, 1996b). In marine invertebrates, these confounding effects of parental care are often absent and eggs or embryos are frequently released into the water column or laid on the benthos, with no additional parental care (Pechenik, 1999; Thorson, 1950). Furthermore, offspring size varies widely among marine invertebrates, even among closely related species (e.g., Allen & Podolsky, 2007; Collin, 2003) and sometimes even within a species (Zakas & Rockman, 2014). Across broadcast spawning marine invertebrates, 85% of species produce planktotrophic larvae, characterized by small egg size and the requirement for offspring to feed on exogenous resources to complete development (Thorson, 1950). In contrast, 10% of marine invertebrates produce lecithotrophic larvae that come from small clutches of large eggs, and rely solely on energy in the yolk to get through early developmental stages (Thorson, 1950). The remaining 5% of species have intermediate development modes (Thorson, 1950), though intermediates may be underreported (Allen & Pernet, 2007).

The wide range of offspring sizes in broadcast spawning marine invertebrates and the lack of parental care following spawning have facilitated the use of a series of influential optimality models to explain theoretical trade‐offs between clutch size and offspring size (Roff, 1992; Sinervo, 1990; Smith & Fretwell, 1974; Stearns, 1992; Vance, 1973). These models generally assume that there is a single optimal egg size for each species, and many predict that egg size extremes are favored (e.g., Vance, 1973). While very influential in life history theory, optimality models are only as good as the empirical data that underlie them. Among marine invertebrates, the richest empirical data sets come from echinoderms (sea urchins, sea stars, etc; Emlet et al., 1987; Levitan, 2000; McEdward & Janies, 1993; Sinervo & McEdward, 1988; Strathmann, 1987). Echinoderms are a useful model for studying the effects of variable offspring provisioning because they demonstrate a variety of developmental modes among close relatives and generally lack parental care after gametes have been spawned. Additionally, among planktotrophic echinoderms, the mean egg energy content is significantly correlated with the mean egg volume, suggesting that egg size is a good predictor of egg energy content across species (Jaeckle, 1995; McEdward & Chia, 1991) with some important limitations (McEdward and Morgan, 2001; Moran & McAlister, 2009). The regulative development of echinoderms also allows embryo energy content to be directly manipulated through blastomere separations or deletions, which can experimentally mimic evolutionary transitions in egg size (e.g., Emlet et al., 1987; Horstadius, 1973; Jenkinson, 1909; Sinervo & McEdward, 1988; Wray, 1999).

In echinoids, the best studied group of echinoderms, planktotrophic larvae that develop from experimentally reduced embryos are viable, but smaller and take longer to develop than those that develop from whole embryos (Alcorn & Allen, 2009; Allen, 2012; Sinervo & McEdward, 1988). For example, in the congeneric echinoid species *Strongylocentrotus purpuratus* and *S. droebachiensis*, larvae from half‐embryos are smaller, have a simpler body plan, and develop more slowly than those from whole embryos (Sinervo & McEdward, 1988). Hart (1995) found that blastomere separations in *S. droebachiensis* also reduce juvenile size. Alcorn and Allen (2009) and Allen (2012) found that embryo energy reductions both extend larval development time and reduce juvenile size in several species of planktotrophic echinoids (*Arbacia punctulata*, *Echinarachnius parma*, *Dendraster excentricus*, *S. droebachiensis*, and *S. purpuratus*) across a range of egg sizes and levels of per‐offspring investment.

While the work performed on echinoids is extensive, it is taxonomically narrow and tends to overlook the potential importance of latent effects that are now well known in a number of marine invertebrate phyla (reviewed in Pechenik, 2006 and Pechenik, 2018). In a rare example of embryo reductions in a non‐echinoderm marine invertebrate, Pernet, Amiel, and Seaver (2012) experimentally reduced embryo size in the marine annelid, *Capitella teleta*. Using laser ablation of macromeres, Pernet et al. (2012) detected significant reductions in larval length and juvenile size when embryonic energy was reduced, consistent with results from echinoids, and suggesting that in lecithotrophic species, maternal energy is allocated for the formation of large, high‐quality juveniles (Pernet et al., 2012). Our goal is to test whether the patterns described in echinoids, and now one species of annelid, hold across other echinoderm species by examining the relationship between egg size, embryo energy, and development in two species of sea stars (Echinodermata: Asteroidea).

Much like the methods for reducing embryo energy in echinoids, experimental embryology can be used to study the effects of reductions in embryo energy content in asteroids. Blastomeres isolated from 2‐, 4‐, and 8‐cell sea star embryos have been shown to develop into morphologically normal larvae, although smaller in size (Dan‐Sohkawa & Satoh, 1978). In the present study, we examined the effects of food concentration and embryo energy reductions on the larval and juvenile development of two planktotrophic species of asteroids: *Pisaster ochraceus* and *Asterias forbesi*. *P. ochraceus* eggs are between 150 and 180 µm in diameter, while *A. forbesi* eggs are between 110 and 140 µm in diameter (Emlet et al., 1987). Among planktotrophic developers, the average egg size is 150 µm, so *P. ochraceus* eggs are slightly larger than the average planktotroph and *A. forbesi* eggs are slightly smaller. For each of these species, we examined the effects of embryo energy reductions on the development under high larval food conditions. For *A. forbesi*, we then repeated our initial experiment while adding in a manipulation of larval food level (low and high) to test the effects of embryo energy reductions when exogenous food is limiting. Furthermore, because *A. forbesi* produces eggs that vary up to twofold in volume within a single clutch (Blackburn, 2013), we also investigated the effects of natural intraclutch variation in egg size and its interaction with food concentration on larval and juvenile development. The natural intraclutch variation in egg size allowed us to compare these results with those from experimental manipulations of embryo energy. These experiments significantly extend our understanding of how maternal investment and larval food environment influence juvenile quality in marine invertebrates with complex life cycles.

## 2 METHODS

### 2.1 Adult collection

In summer 2014, adult *P. ochraceus* were collected from Snug Harbor on San Juan Island, Washington (48°34′21″N, 123°10′19″W), and transported to Friday Harbor Laboratories, Friday Harbor, WA where they were kept in flow‐through sea tables at ambient salinity (22–31 ppt) and temperature (10–16°C). Adult *A. forbesi* were obtained in fall 2014 from the Marine Biological Laboratory, Woods Hole, MA and shipped overnight to the College of William and Mary in Williamsburg, VA, where they were maintained in aquaria with recirculating artificial seawater (ASW; Instant Ocean, Spectrum Brands, Blacksburg, VA) at 32 ppt and 12–14°C. In the summers of 2015 and 2016, adult *A. forbesi* were collected by snorkel from shallow (1–5 m) subtidal habitats at Rockland Breakwater, Rockland, Maine (44°6′47″N, 69°04′52″W), and transported to Bowdoin College Schiller Coastal Studies Center on Orr's Island, Maine, where they were kept in flow‐through sea tables at ambient salinity (29–33 ppt) and temperature (13–18°C).

### 2.2 Experiments 1 and 2: manipulation of embryo energy

Trials were conducted with *P. ochraceus* during summer 2014 and with *A. forbesi* during spring 2015 to test the effects of embryo energy reductions on larval and juvenile development. For each species, adults were induced to spawn through an intracoelomic injection of 100 µM 1‐methyladenine (9 ml for *P. ochraceus*; 3 ml for *A. forbesi*). One male/female pair was crossed for each trial. In order to allow sufficient time for embryonic manipulations following first cleavage, we conducted staggered fertilizations every 30 min for 2 hr. For each fertilization, batches of several thousand eggs were gently pipetted into glass bowls containing 150 ml of 0.45 µm filtered seawater (FSW; *P. ochraceus* trial) or ASW (*A. forbesi* trial) and were then fertilized with 1 ml of dilute sperm. At each time point, a subsample of 50 eggs was scored for fertilization to ensure high (>90%) fertilization success. Preliminary testing suggested that within a 2‐hour window, there were no negative effects of delayed fertilization on development in either species.

At the first visible signs of cleavage (~4 hr postfertilization for *P. ochraceus* and ~2 hr postfertilization for *A. forbesi*), 40–50 embryos at the two‐cell stage were placed on glass slides in a minimal volume of water (<200 µl) and randomly designated to either receive a laser ablation treatment or to serve as a control. A footed coverslip was created by adhering fragments of a #1.5 coverslip to the edges of a #1.5 coverslip with melted dental wax, which was then placed over the embryos to prevent evaporation. Embryos in the laser treatment were placed under a microscope with a Hamilton Thorne XYClone infrared laser mounted on a 20× objective. The laser was fired at one blastomere of each two‐cell embryo at 100% power for 100 ms. Based on a pilot study, 100 ms was the minimum amount of time needed to kill the cell and puncture the fertilization envelope (FE). The result of puncturing the FE was that most or all of the cytoplasm from the killed cell leaked out of the FE and was therefore unavailable to be reabsorbed by the remaining, still living, blastomere. After all embryos on a single slide were treated with the laser, embryos were rinsed into 5‐cm‐diameter petri dishes containing approximately 10 ml FSW or ASW. In order to control for any effects of sitting at room temperature in a small volume of water, slides with control embryos were left on the countertop until the laser treatment was completed, at which point control embryos were also rinsed into separate petri dishes.

After allowing embryos to develop in petri dishes for 24 hr, larvae were transferred into 250‐mL beakers at a density of 1 larva 10 ml^−1^. The *P. ochraceus* trial had five replicate beakers for each treatment, while the *A. forbesi* trial had nine replicate beakers per treatment. All beakers were placed on a stirring rack with a motor to stir paddles at a rate of 10 strokes/min (Strathmann, 1987). Beaker position was rotated every other day to account for any potential effects of position within the stirring rack. Beaker cleaning and water changes were completed every other day by reverse filtering 50% of the water in each beaker through 35‐µm mesh and then refilling beakers to 200 ml with either FSW or ASW. After water changes, larvae were fed a combination of three species of algae: *Dunalliela tertiolecta* (UTEX Culture Collection of Algae, Austin TX, Catalog #LB999), *Isochrysis galbana* (National Center for Marine Algae and Microbiota, West Boothbay Harbor, ME, Catalog #CCMP1323), and *Rhodomonas lens* (National Center for Marine Algae and Microbiota, West Boothbay Harbor, ME, Catalog #CCMP739). Each beaker received 7,500 algal cells species^−1^ ml^−1^. Mean rearing temperature for Experiment 1 was ~12.5°C and for Experiment 2 was ~18°C.

When larvae began to develop a juvenile rudiment and brachiolar arms, a blue mussel shell (*Mytilus trossulus* for *P. ochraceus* and *Mytilus edulis* for *A. forbesi*) was added to each beaker as a settlement cue and beakers were no longer cleaned to promote biofilm establishment on the glass. Water changes and larval feeding protocols continued on alternating days until experiments were completed. Metamorphosed juveniles were removed daily from beakers and isolated in 6‐well plates. Larval development time was recorded for each individual, and juvenile area and the number of spines were recorded 2 days postmetamorphosis. Photographs of each juvenile were also taken 2 days postmetamorphosis using a Canon DSLR camera attached to an Olympus CX 41 microscope at a total magnification of 400×. From these photographs, area was later measured in ImageJ64 (Rasband, W.S., ImageJ, U. S. National Institutes of Health, Bethesda, Maryland, USA, https://imagej.nih.gov/ij/, 1997–2016.). The proportion of larvae reaching metamorphosis was also recorded for each beaker. Disk area, disk diameter, and spine number for *P. ochraceus* juveniles were remeasured between 72 and 73 days and then again at 108 days postfertilization. Survival was recorded at 138 and 172 days postfertilization. *A. forbesi* juveniles were remeasured 20 days postmetamorphosis, and survival was not tracked after this time.

### 2.3 Experiment 3: manipulation of embryo energy and food supply

Experiments 1 and 2 were repeated for *A. forbesi*, with an additional manipulation of food concentration, in order to determine the importance of endogenous energy reserves versus exogenous energy resources in sea star development. This experiment was a 2 × 2 fully crossed design manipulating egg size and food concentration. Spawning and treatment methods were identical to the methods of Experiment 1 with the exception that beakers from the laser and control treatments were randomly assigned either a low food concentration (2,500 algal cells algal species^−1^ ml^−1^) or a high food concentration (7,500 algal cells algal species^−1^ ml^−1^). There were 46 beakers in total: 11 laser/high food treatment, 11 control/high food treatment, 12 laser/low food treatment, and 12 control/low food treatment. Larval rearing methods were identical to the methods described in Experiment 1, except the rearing temperature was ~16.5°C. The proportion of larvae reaching settlement, larval development time, spine number, and area were measured as described previously. Juveniles were remeasured 20 days postmetamorphosis, and survival was tracked until death.

### 2.4 Experiment 4: investigating natural intraclutch variation in egg size in *A. forbesi*

After investigating experimental reductions of embryo size, we examined how natural variation in egg size affects larval development in *A. forbesi*. As in experiment 3, we simultaneously manipulated larval food concentration to determine the importance of endogenous energy reserves and exogenous energy resources during the development of larvae. Therefore, this was a 2 × 2 fully crossed design manipulating egg size and food concentration.

Two females with natural intraclutch egg size variation were used, with larvae from each female reared separately. For each female, unique males were used to fertilize the eggs. After spawning adults and fertilizing eggs as described in Experiment 1, large and small embryos were separated by hand under a dissecting microscope and their diameter was measured under a compound microscope to confirm egg size differences. Each beaker containing either 20 large embryos or 20 small embryos was randomly assigned a larval food concentration of low (1,000 algal cells algal species^−1^ ml^−1^) or high (7,500 algal cells algal species^−1^ ml^−1^). For each female, there were five beakers in each of the four treatments, cultured at ambient temperature (~17°C). All other aspects of larval rearing were identical to previous experiments. Our response variables for this experiment were survival to settlement, age at settlement, juvenile area, and juvenile spine number in each treatment.

### 2.5 Data analysis

The data analysis for these experiments was completed in IBM SPSS Statistics (version 23). In experiments 1 and 2, a mixed‐model ANOVA with laser treatment as a fixed factor and beaker as a random factor was used to evaluate the following response variables: survival to settlement, age at settlement, juvenile area, and juvenile spine number. We tested residuals for normality using Kolmogorov–Smirnov and Shapiro–Wilk tests. We used a mixed‐model ANCOVA (with age as the covariate) on the proportion of juveniles alive at each time point from each treatment to determine whether treatment influenced mortality. Proportions were arcsine square‐root‐transformed prior to analysis. We also conducted a nonparametric alternative, a rank transformation ANCOVA, because the data violated assumptions of the parametric ANCOVA (Olejnik & Algina, 1984; Quade, 1967). While the rank transformation ANCOVA is an accepted nonparametric alternative to an ANCOVA, there are concerns with the tests’ ability to detect heterogeneity of slopes in ANCOVA designs (Quinn & Keough, 2002). Therefore, we felt it appropriate to include both the parametric ANCOVA and nonparametric alternative (the rank transformation ANCOVA) in the analysis and results.

The data analysis for experiment 3 was identical to the analysis in experiments 1 and 2 with the addition of food treatment and the interaction of food and laser treatments as fixed factors in the linear mixed‐model ANOVA. The analysis of mortality was conducted using identical methods as described above with the addition of food treatment as a fixed factor in the mixed‐model ANCOVA and the rank transformation ANCOVA.

To analyze the data from experiment 4, we used a linear mixed‐model ANOVA to evaluate the response variables’ survival to settlement, age at settlement, juvenile area, and juvenile spine number. For all response variables, female was modeled as a random factor with egg size and larval food treatments as fixed factors. Residuals were tested for normality using the Kolmogorov–Smirnov and Shapiro–Wilk tests.

## 3 RESULTS

### 3.1 Experiments 1 and 2: manipulation of embryo energy

**FIGURE 1:**
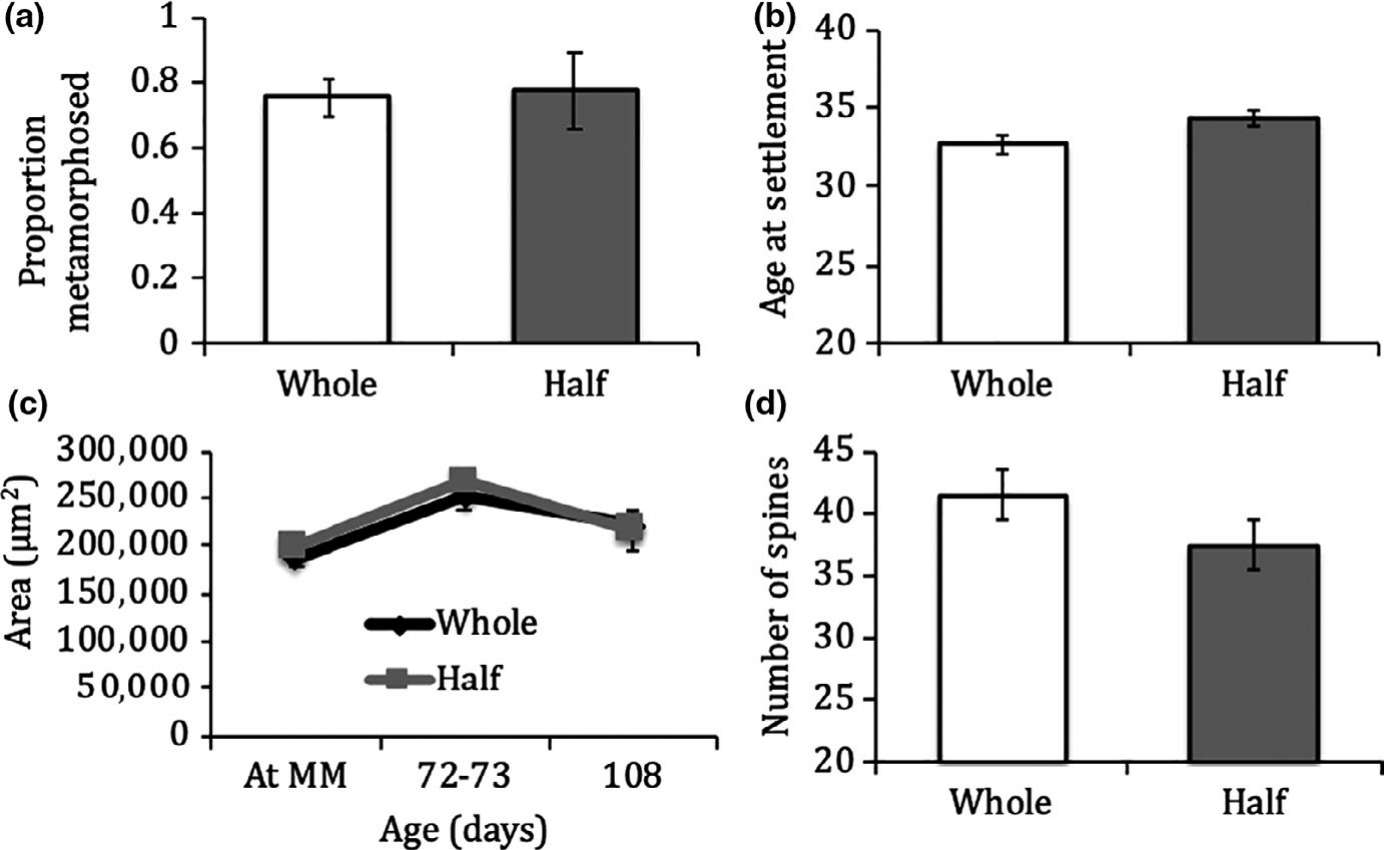
Average proportion metamorphosed (a), age at settlement (b), disk area (c), and number of spines (d) for *Pisaster ochraceus*. Unshaded bars represent control embryos; shaded bars represent laser‐treated embryos. Bars are means ± *SE*

In experiment 1, *P. ochraceus* juveniles began settling 30 days postfertilization and, across all beakers, 76% of larvae reached metamorphosis (75% of whole embryos reached metamorphosis, and 77% of half‐embryos reached metamorphosis; Figure 1a). Based on a linear mixed‐model ANOVA, no significant differences were detected between offspring from whole (control) and half (laser‐ablated) embryos in any of the following response variables: proportion surviving to metamorphosis, age at metamorphosis, area at metamorphosis, or spine number (Figure 1a–d; Table 1A–D). While, as predicted, larvae from whole embryos metamorphosed sooner than larvae from half‐embryos, the 5% increase in development time for laser‐ablated embryos was not significant (Figure 1b; Table 1B). Also in line with our predictions, juveniles from half‐embryos had fewer spines compared to juveniles from whole embryos; however, this 9.7% reduction was also not significant (Figure 1d; Table 1D). In addition to measures at metamorphosis, we also tracked starved juveniles postmetamorphosis to test for delayed effects of embryo reductions on size or survival. There was no significant effect of embryo size on the areas of *P. ochraceus* juveniles 72–73 days postfertilization (Figure 1c; Table 1E) or 108 days postfertilization (Figure 1c; Table 1F). There was also no significant effect of embryo size on the survival of *P. ochraceus* juveniles postmetamorphosis (linear mixed‐model ANCOVA: *F*_1,56_ = 0.287, *p* = .594; rank transformation ANCOVA: *F*_1,58_ = 3.023, *p* = .087). All juveniles, regardless of embryo size treatment, were deceased by 172 days postfertilization.

**TABLE 1:** A linear mixed‐model ANOVA on the effects of laser treatment on proportion metamorphosed, age at settlement, area, spine number, second measured area, and third measured area in *Pisaster ochraceus* juveniles

Predictor	*df*	*F*‐value	*p*‐value
(A) Proportion metamorphosed	1, 8	0.006	.941
(B) Age at settlement	1, 8	4.191	.075
(C) Area	1, 8	0.911	.368
(D) Spine number	1, 8	0.806	.396
(E) Second area measurement	1, 8	0.429	.531
(F) Third area measurement	1, 8	0.067	.802

**FIGURE 2:**
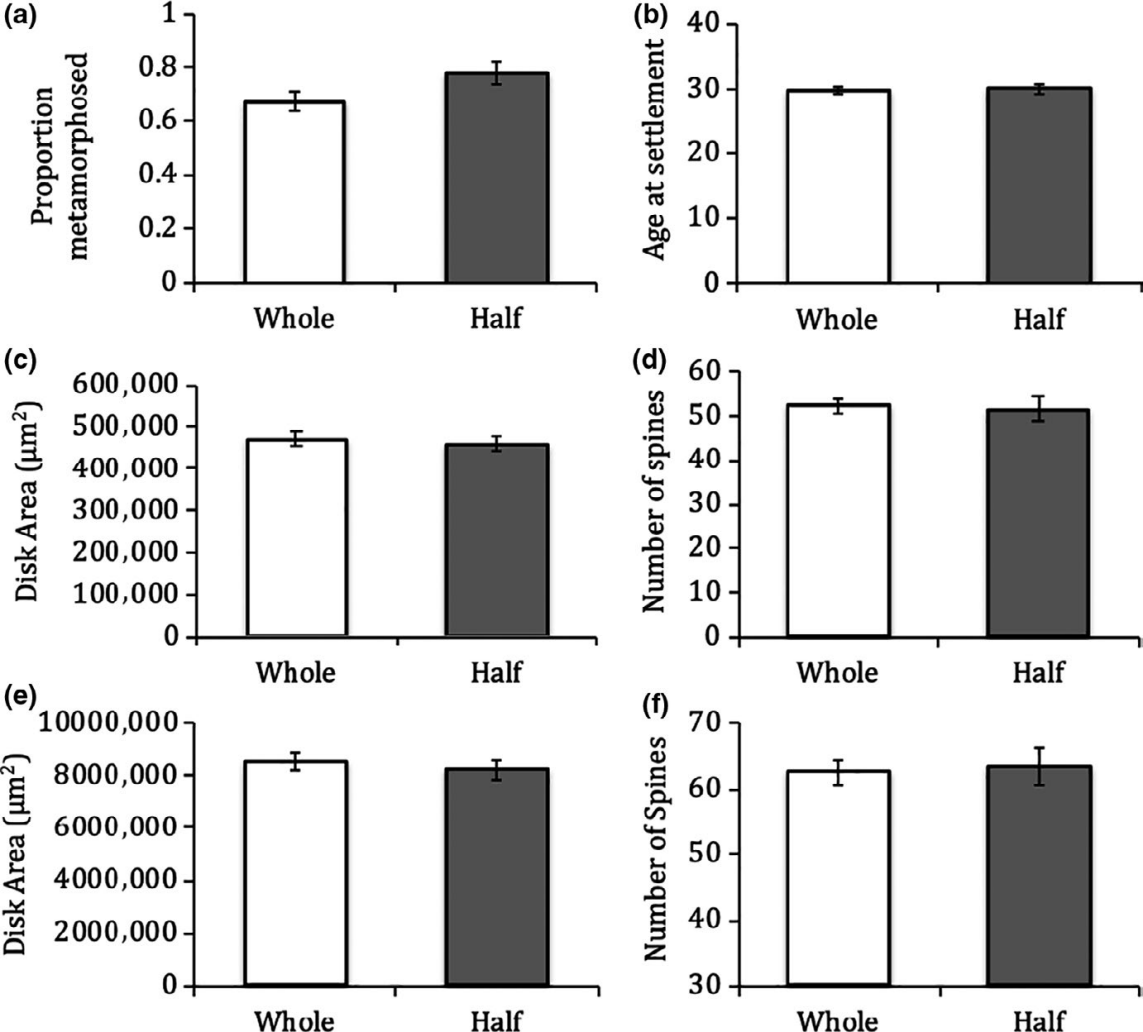
Average proportion metamorphosed (a), age at settlement (b), disk area (c), number of spines (d), disk area 20 days post‐MM (e), and number of spines 20 days post‐MM for *Asterias forbesi*. Unshaded bars represent control embryos; shaded bars represent laser‐treated embryos. Bars are means ± *SE*

In experiment 2, *A. forbesi* juveniles began settling 22 days postfertilization with 72.5% of all larvae reaching metamorphosis across all beakers (78% of half‐embryos and 67% of whole embryos reached metamorphosis). There was no significant difference in the proportion of larvae metamorphosing between those from whole and half‐embryos (Figure 2a; Table 2A). *Asterias forbesi* also exhibited no differences between embryo size treatments in the age at metamorphosis, area, or spine number at metamorphosis based on a linear mixed‐model ANOVA (Table 2B–D). The lack of a significant effect of embryo size on the area or spine number of *A. forbesi* juveniles persisted through 20 days postmetamorphosis (Figure 2e,f; Table 2E,F).

**TABLE 2:** A linear mixed‐model ANOVA on the effects of laser treatment on proportion metamorphosed, age at settlement, area, spine number, area 20 days postmetamorphosis (MM), and spine number 20 days post‐MM in *Asterias forbesi*

Predictor	*df*	*F*‐value	*p*‐value
(A) Proportion metamorphosed	1, 16	3.628	.075
(B) Age at settlement	1, 16	0.136	.717
(C) Area	1, 16	0.234	.635
(D) Spine number	1, 16	0.059	.826
(E) Area 20 days post‐MM	1, 16	0.402	.535
(F) Spine number 20 days post‐MM	1, 16	0.141	.712

### 3.2 Experiment 3: manipulation of embryo energy and food supply

**FIGURE 3:**
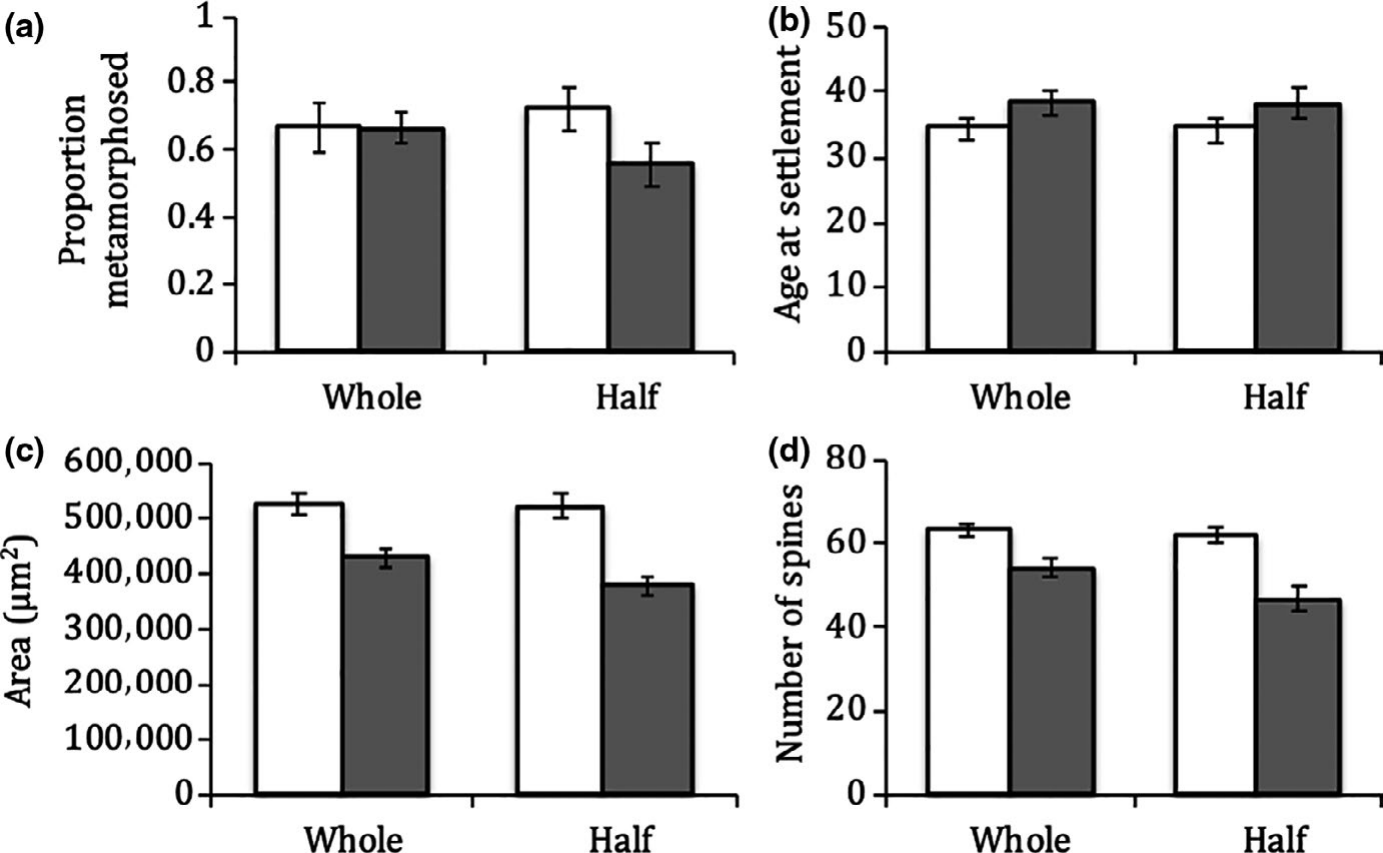
Average proportion metamorphosed (a), age at settlement (b), disk area (c), and number of spines (d) for *Asterias forbesi*. Unshaded bars represent high food‐treated larvae (7,500 algal cells algal species^−1^ ml^−1^); shaded bars represent low food‐treated larvae (2,500 algal cells algal species^−1^ ml^−1^). Bars are means ± *SE*

Juveniles began settling 23 days postfertilization, with 65% of all larvae reaching metamorphosis (Figure 3a). There was no significant effect of larval food treatment, embryo size treatment, or their interaction on the percent of larvae reaching metamorphosis (Table 3A). There was an 11% increase in settlement age between larvae fed low food concentrations and larvae fed high food concentrations; however, these results were not significant (Figure 3b; Table 3B). Similarly, there was no significant effect of embryo size treatment or the interaction of embryo size and food treatment on the settlement age of *A. forbesi* larvae (Table 3B). There was a 23% increase in the area of *A. forbesi* juveniles at metamorphosis due to increased larval food (Figure 3c; Table 3C). There was no significant effect of embryo size treatment or the interaction of food and embryo size treatments on the area of *A. forbesi* juveniles at metamorphosis (Table 3C). There was a 20% increase in spine number due to increased larval food (Figure 3d; Table 3D), but no significant effect of embryo size treatment or the interaction of embryo size and food treatments on the spine number of *A. forbesi* juveniles at metamorphosis (Table 3D).

**TABLE 3:** A linear mixed‐model ANOVA on the effects of laser treatment, food treatment, and the interaction of laser and food treatments on proportion metamorphosed, age at settlement, area, and spine number in *Asterias forbesi*

Predictor	*df*	*F*‐value	*p*‐value
(A) Proportion metamorphosed			
Food	1, 41	2.201	.146
Embryo size	1, 41	0.062	.805
Food × Embryo size	1, 41	2.131	.152
(B) Age at settlement			
Food	1, 41	3.961	.053
Embryo size	1, 41	0.001	.979
Food × Embryo size	1, 41	0.004	.951
(C) Area			
Food	1, 41	42.925	**<.001**
Embryo size	1, 41	1.967	.168
Food × Embryo size	1, 41	1.557	.219
(D) Spine number			
Food	1, 41	27.932	**<.001**
Embryo size	1, 41	3.712	.061
Food × Embryo size	1, 41	1.728	.196

Note

Bold signifies significant values (p < 0.05)

All juveniles, regardless of treatments, were deceased by 120 days postmetamorphosis. Food treatment had a significant effect on the rate of death of *A. forbesi* juveniles with juveniles from larvae fed low food treatments dying sooner than juveniles from larvae fed high food concentrations (linear mixed‐model ANCOVA: *F*_1,502_ = 4.428, *p* = .036; rank transformation ANCOVA: *F*_1,502_ = 15.326_,_ *p* < .001). There was no significant effect of embryo size treatment (linear mixed‐model ANCOVA: *F*_1,502_ = 0.147, *p* = .701; rank transformation ANCOVA: *F*_1,502_ = 1.648, *p* = .200) or the interaction of embryo size and food treatments (linear mixed‐model ANCOVA: *F*_1,502_ = 0.066, *p* = .797; rank transformation ANCOVA: *F*_1,502_ = 0.110, *p* = .741) on the age at death of *A. forbesi* juveniles.

### 3.3 Experiment 4: investigating natural intraclutch variation in egg size in *A. forbesi*

Egg sizes for the first female were 117 ± 4.4 µm and 133 ± 11.23 µm (small and large), respectively, reflecting a 1.47‐fold difference in volume. Egg sizes for the second female were 112 ± 6.6 µm and 151 ± 6.9 µm (small and large), respectively, reflecting a 1.99‐fold difference in volume. Across both females, there was a 1.70‐fold difference in egg size overall.

**FIGURE 4:**
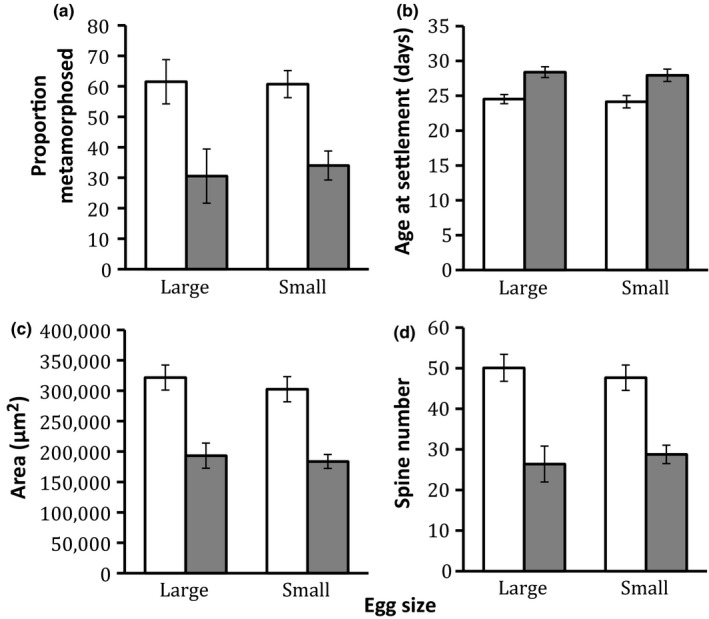
Average proportion metamorphosed (a), age at settlement (b), disk area (c), and number of spines (d) for *Asterias forbesi*. Unshaded bars represent high food‐treated larvae (7,500 algal cells algal species^−1^ ml^−1^); shaded bars represent low food‐treated larvae (1,000 algal cells algal species^−1^ ml^−1^). Bars are means ± *SE*

Across all beakers, 46% of larvae survived to settlement, with 32.4% survival among larvae reared in the low larval food treatment and 61.2% survival among larvae reared in the high larval food treatment (Figure 4a). There was no effect of egg size on the percent of larvae surviving to metamorphosis (Table 4A). There was, however, a significant effect of larval diet on the proportion of larvae surviving to metamorphosis (Table 4A) such that larvae reared on high food treatment had greater survival to metamorphosis (Figure 4a). Larvae reared on low food took an average of 4.4 days longer to reach metamorphosis compared to larvae reared on high food (15.3% increase; Table 4B), but egg size had no significant effect on age at metamorphosis (Figure 4b, Table 4B). Juveniles from the low food treatment also had a significantly smaller area (43.5% decrease; linear mixed model: *F*_1,30_ = 42.043, *p* < .001) and significantly fewer spines (45.4% decrease; linear mixed model: *F*_1,30_ = 35.495, *p* < .001 than did larvae in the high food treatment (Figure 4c,d, Table 4C,D). Egg size had no significant effect on juvenile area (linear mixed model: *F*_1,30_ = 0.032, *p* = .859) or juvenile spine number (Figure 4c,d, Table 4C,D). For all response variables, the interaction between larval food treatment and egg size was not significant (Table 4).

**TABLE 4:** A linear mixed‐model ANOVA on the effects of egg size, food treatment, and the interaction of egg size and food treatments on proportion metamorphosed, age at settlement, area, and spine number in *Asterias forbesi*

Predictor	*df*	*F*‐ratio	*p*‐value
(A) Proportion metamorphosed			
Food	1, 30	24.723	**<.001**
Egg size	1, 30	0.032	.859
Food × Egg size	1, 30	1.598	.216
(B) Age at settlement			
Food	1, 30	39.262	**<.001**
Egg size	1, 30	0.232	.633
Food × Egg size	1, 30	0.354	.557
(C) Area			
Food	1, 30	42.043	**<.001**
Egg size	1, 30	0.081	.778
Food × Egg size	1, 30	1.051	.313
(D) Spine number			
Food	1, 30	35.495	**<.001**
Egg size	1, 30	0.429	.518
Food × Egg size	1, 30	0.193	.664

Note

Bold signifies significant values (p < 0.05)

## 4 DISCUSSION

For the two planktotrophic asteroids in this study, larval food concentration was much more important than offspring size in determining age at metamorphosis, juvenile size, and juvenile spine number. While changes in larval food concentration had significant effects on all of these traits, changes in offspring size had relatively weak and statistically insignificant effects on larval and juvenile development. These results appear to differ from previous results from echinoids, which consistently show that reduced egg size significantly increases development time and decreases juvenile size (Alcorn & Allen, 2009; Allen, 2012; Hart, 1995; McEdward, 1996; Sinervo & McEdward, 1988). Care should be taken in using results from echinoids as a paradigm for marine invertebrate responses to reductions in maternal investment, as our work shows this paradigm does not hold true even in other echinoderm classes. Our results also have implications for life history theory and egg size evolution more generally. For example, if changes in offspring size have only weak effects on development time and juvenile performance, it is likely there are other selective pressures on asteroids that have shaped the evolution of maternal investment.

Previous reductions of embryo energy content in planktotrophic echinoids resulted in an increase in larval development time (Alcorn & Allen, 2009; Allen, 2012; McEdward, 1996; Sinervo & McEdward, 1988) comparable to the inversely proportional relationship between egg size and development time derived from comparative data sets (Levitan, 2000). Based on embryo energy reductions in seven species of echinoids (figure 7 in Allen, 2012), we predicted a 9.7% increase in development time in *P. ochraceus* and an 11.1% increase in development time in *A. forbesi* when embryo energy is reduced by 50%. However, we found that changes in embryo energy content did not significantly affect the age at metamorphosis in either species, yielding increases of only 5% in *P. ochraceus* and 1.3% in *A. forbesi*. We conducted a power analysis to determine the sample size we would have needed to detect a significant increase in development time in *P. ochraceus* and in *A. forbesi*. If the magnitude of the effects of embryo energy reductions had aligned with predictions based on previous studies in echinoids, the power of Experiments 1, 2, and 3 to detect these differences was >0.99 in each case. Given the extremely high powers associated with our experiments, we conclude that there simply does not appear to be an effect of embryo energy reductions on larval development time in *P. ochraceus* or *A. forbesi*. Despite this, our experimental design was, however, limited in other ways. First, we had limited biological replication, reporting results from only a single male/female pair for three of our four experiments. Population genetic differences are thus not well represented in our data set and may explain some of the differences between the high food treatments reported across *A. forbesi*. In addition to genetic differences, there were also differences in larval culture temperature and seawater (natural vs. artificial) that may explain differences across experiments (Pechenik, 1999).

We also found no significant developmental differences in *A. forbesi* larvae reared from small eggs versus larvae reared from eggs that were naturally 1.7 times greater in volume. We predicted that offspring from large eggs would develop to metamorphosis sooner and that juveniles would be larger with more spines. However, offspring quality was the same regardless of egg size. One possible explanation for this result is that the size of the eggs does not reflect energy content. This would be in contrast to previous studies that have shown that egg size strongly correlates with egg energy content (Jaeckle, 1995). However, the differences in egg size within a clutch may be due to imprecision in gametogenesis or an adaptive response to variation in the environment, such as larval food, instead of differences in the allocation of resources (McEdward & Carson, 1987) as has been suggested to explain poor correlations between egg size and embryo energy in other invertebrates (Moran & McAlister, 2009).

While we found no significant developmental effects of embryo energy reductions in any of our experiments, we found that larval food concentration had significant effects in *A. forbesi*. In previous experiments, echinoderm larvae reared at lower food concentrations resulted in fewer metamorphs than larvae reared at higher food concentrations (Allen, 2012; Uthicke, Schaffelke, & Byrne, 2009). We found the same results in one of two experiments (Experiment 4) that manipulated larval food concentration. A possible explanation for the lack of significant differences in larval success between low and high food treatments in Experiment 3 is that the low food concentration level was 2.5× higher in Experiment 3 than in Experiment 4. Many asteroid species, including *P. ochraceus* and *A. forbesi*, exhibit phenotypic plasticity in limited food environments by growing longer larval arms that allow them to increase clearance of food particles in the water column (George, 1999; Richardson, 2018; Wolfe, Graba‐Landry, Dworjanyn, & Byrne, 2015). Phenotypic plasticity in low food environments has also been observed in other echinoderm larvae including echinoids and ophiuroids, with organisms increasing the size of their feeding structures when exposed to low food environments (e.g., Hart & Strathmann, 1994; Podolsky & McAlister, 2005; Miner, 2007; see McAlister & Miner, 2018 for a review). Phenotypic plasticity in ciliated band length may allow for some degree of compensatory growth among planktotrophic asteroids. While arm length was not directly measured in our experiments, our finding of effects of larval food on survival to and time to metamorphosis in Experiment 4 but not Experiment 3 (where “low” food was 2.5× higher) is consistent with partial compensatory growth in sea star larvae mediated by larval arm plasticity.

To further support the idea that asteroids have stronger responses to variation in larval food compared to variation in embryo size, we found that embryo size had no significant effect on juvenile area, juvenile spine number, or survival in *P. ochraceus* and *A. forbesi*. However, we did find food concentration significantly affected juvenile area at metamorphosis in *A. forbesi*. Based on an analysis of embryo energy reductions in seven species of echinoids (figure 7 in Allen, 2012), we predicted a 10.5% decrease in juvenile size in *P. ochraceus* and a 10.1% decrease in juvenile size in *A. forbesi* when embryo energy was reduced by 50%. However, we found no significant effects of embryo energy on juvenile size in either species. A decreased size at metamorphosis can have negative consequences for asteroid juveniles, including a weakened attachment to the substrate or a decrease in the body calcification of the juvenile, making juveniles more vulnerable to predators (Basch & Pearse, 1996). For example, differences in juvenile energy content at metamorphosis, which is correlated with juvenile size, in the planktotrophic sea star, *Acanthaster planci*, had significant effects on the behavior, growth, and physiology of the juveniles (Zann, Brodie, Berryman, & Naqasima, 1987). Similarly, in a second planktotrophic species, *Asterina miniata*, smaller juvenile size can affect juvenile survival in low food environments postmetamorphosis (Basch & Pearse, 1996). Instead, given the large negative effect of decreased larval food concentration on the area of juveniles (40% decrease relative to high food) at metamorphosis in *A. forbesi*, it is likely that larval food concentration plays a much more prominent role in the success of juveniles postmetamorphosis than does maternal investment.

Our study investigated three potential costs of decreased embryo size in asteroids: changes in time to metamorphosis, changes in juvenile size (quantified as juvenile area and spine number), and changes in survival postmetamorphosis. There may be other costs of smaller egg and embryo sizes in these species, including increased predation rates and lower fertilization in smaller eggs (Hart, 1995); however, we did not test for these differences. Future experiments could determine whether changing the initial egg size either experimentally or through natural variation leads to differences in these parameters. There may be both costs and benefits to smaller larval size in regard to predation; previous work has shown there may be a higher predation on smaller larvae; however, larger larvae may be preferentially consumed by visual predators (Allen, 2008). Much like changes in predation rate, smaller eggs may exhibit both benefits and costs to the fertilization rate; there is evidence for lower rates of fertilization in smaller eggs (Levitan, 1993); however, species with smaller eggs may avoid this cost of egg size through accessory structures such as jelly coats (Podolsky, 2001) that can allow resilience of fertilization under variable environmental conditions (Podolsky, 2004; reviewed by Deaker, Foo, & Byrne, 2019). One cost of small egg size that has rarely been considered is its potential influence on rates of larval cloning. Asteroid larvae are well known to clone (Bosch et al., 1995; Jaeckle, 1995; Vickery and McClintock, 2000; Knott et al., 2003; Allen et al., 2019; Janies et al., 2019), and their propensity to clone may be related to endogenous energy reserves reflected in the size of the egg, with larger eggs possessing greater reserves and possibly more likely to produce larvae that clone.

In addition to future studies investigating further costs of smaller egg size in these species, future studies should examine the effects of reductions in egg size across additional species with both planktotrophic and lecithotrophic development. The planktotrophic nature of larvae from small eggs may allow compensation for decreases in embryo size by increasing ciliated band length while feeding in the water column (McAlister & Miner, 2018). However, compensatory growth is not possible in lecithotrophic development as these species lack the necessary structures to feed while in the water column and thus cannot compensate for a smaller size through additional energy gain. Therefore, when embryo energy is reduced, lecithotrophic species should exhibit no differences in larval time, but should be smaller in size at metamorphosis (Allen, 2012). For example, juveniles of the lecithotrophic sea urchin, *Heliocidaris erythrogramma*, are smaller at metamorphosis when developing from eggs with reduced lipids; however, they reach metamorphosis at the same time as control larvae (Emlet & Hoegh‐Guldberg, 1997). Similarly, in the facultative planktotroph *Clypeaster rosaceus*, when embryo energy is reduced, juveniles are significantly smaller, but exhibit no detectable effects of energy reduction on larval development time (Allen, Zakas, & Podolsky, 2006). These results indicate embryo energy may have different functions in feeding and nonfeeding larvae, with facultative planktotrophs responding to embryo size reductions more similarly to lecithotrophs than planktotrophs (Allen et al., 2006; Emlet & Hoegh‐Guldberg, 1997). These experimental results support and strengthen models of the relationship between egg size, larval development time, and juvenile size based on comparative data sets in echinoids (Emlet et al., 1987; Levitan, 2000). It would be a useful future experiment to test whether lecithotrophic asteroids respond to decreases in embryo energy similarly to echinoids, indicating similar selection pressure on the evolution of egg size across lecithotrophic species. If, however, lecithotrophic asteroids are also found to respond differently to energy reductions than echinoids, it would indicate different selection pressures and therefore different roles of life history parameters like larval time and juvenile size in the evolution of asteroids when compared to echinoids. In addition, long‐term studies of latent effects in both planktotrophic and lecithotrophic species would be useful to determine how embryo energy effects may carry over across life history stages (reviewed by Pechenik, 2018).

A great deal is still unknown about the evolution of egg size and the effects of changing the initial egg/embryo energy content in echinoderms and other marine invertebrates. Experiments similar to ours in taxonomically diverse groups can help to uncover the drivers of egg size evolution in marine invertebrates, and our work suggests that even within a phylum, these drivers are likely to differ.

## CONFLICT OF INTEREST

None declared.

## AUTHOR CONTRIBUTIONS

**Stacy N. Trackenberg:** Conceptualization (supporting); data curation (equal); formal analysis (equal); funding acquisition (supporting); investigation (equal); methodology (equal); project administration (supporting); writing – original draft (equal); writing – review and editing (supporting). **Emily L. Richardson:** Conceptualization (supporting); data curation (equal); formal analysis (equal); funding acquisition (supporting); investigation (equal); methodology (equal); project administration (supporting); writing – original draft (supporting); writing – review and editing (supporting). **Jonathan D. Allen:** Conceptualization (lead); data curation (equal); formal analysis (equal); funding acquisition (lead); investigation (equal); methodology (equal); project administration (lead); resources (lead); software (lead); supervision (lead); writing – original draft (equal); writing – review and editing (lead).

## Data Availability

Data on proportion settling, age at settlement, size, and spine number at settlement are archived on Dryad: https://doi.org/10.5061/dryad.m37pvmczv.
